# Preparation of a Macromolecular Flame Retardant with a Phosphine Oxide Structure and Its Application in Polyamide 6

**DOI:** 10.3390/polym17040475

**Published:** 2025-02-11

**Authors:** Ke Liu, Bohan Liang, Shujuan Zhang, Ruyi Li, Junming Dai, Wangyang Lu

**Affiliations:** 1 National & Local Joint Engineering Research Center for Textile Fiber Materials and Processing Technology, School of Materials Science and Engineering, Zhejiang Sci-Tech University, Hangzhou 310018, China; ruyi_li0311@163.com (R.L.); luwy@zstu.edu.cn (W.L.); 2 Zhejiang Provincial Innovation Center of Advanced Textile Technology, Shaoxing 312000, China; lbh20210319@163.com (B.L.); shujuanzhang@163.com (S.Z.); 13813128598@163.com (J.D.); 3 Shaoxing Keqiao Research Institute, Zhejiang Sci-Tech University, Shaoxing 312000, China

**Keywords:** PA6, phosphine oxide, flame-retardant mechanism

## Abstract

In this study, a novel macromolecular flame retardant (MFR) with a phosphine oxide structure is successfully synthesized to improve the flame retardancy of polyamide 6 (PA6). Following this, the flame-retardant polyamide 6 (FR–PA6) is prepared via melt blending the MFR with PA6. Results indicate that the introduction of MFR has little effect on the melting and crystallization temperature of FR–PA6. While it slightly reduces the thermal stability of PA6, MFR significantly enhances its flame retardancy. The limiting oxygen index of FR–PA6 increases from 21.8% to 28.2%, and it successfully passes the UL-94 V-0 rating when it contains 0.5 wt% of phosphorus. Compared with pure PA6, the av-EHC of FR–PA6 is reduced by 32.2% and the SEA is increased by 66.7%. The MFR showed a flame-retardant mechanism in both the gas phase and the condensed phase. In the gas phase, the decomposition of MFR releases phosphorus-containing free radicals to interrupt the combustion chain reaction and reduces the concentration of the combustible caprolactam. In the condensed phase, the MFR promotes faster formation of melt droplets during combustion, taking heat away from the burning PA6 timely.

## 1. Introduction

Polyamide 6 (PA6), an important engineering thermoplastic, has excellent mechanical properties and chemical corrosion resistance. Therefore, it has been widely used in many domains such as transportation, electronic devices, and home textiles [1,2,3,4]. However, the flammability of PA6 limits its applicability across several domains. Therefore, its flame retardancy must be improved to meet fire safety requirements [5,6,7].

Thus, flame retardancy systems with various chemical structures were studied to improve the flame retardancy of PA6. Halogen flame retardants are well-regarded for their high flame-retardant efficiency; however, some release toxic gases during use and have been banned to meet the requirements for environmental safety. Thus, halogen-free flame retardants have become the mainstream of the future [8,9]. Phosphorus flame retardants are highly efficient halogen-free flame retardants with low toxicity, high flame-retardant efficiency, and a wide application range; they are ideal alternatives for halogen flame retardants [10,11,12,13]. Xiang et al. introduced α-zirconium phosphate (α-ZrP) and ammonium sulfamate (AS) for enhanced flame-retardant properties of PA6. When the α-ZrP content reaches 3 wt%, the LOI value of PA6/2.0AS/3.0α-ZrP increases to 38.7%. The UL-94 rating transforms from V-2 to V-0 when the dosage of α-ZrP is more than 1.5 wt% [14]. Huang et al. synthesized cuprous diethylphosphinate (CuDP) to improve the flame retardancy and thermal oxidative stability of PA6. The introduction of CuDP can significantly improve the fire safety of PA6. PA6 contains 15 wt% CuDP and reaches a high LOI value of 28.2 and passes the UL-94 V-0 rating [15].

However, phosphorus flame retardants have garnered considerable attention as a new pollutant in recent years due to increasing environmental emissions. Most existing phosphorus flame retardants have low molecular weight and are easy to precipitate, causing water pollution and serious biological hazards. Zhang et al. analyzed the concentrations and removal efficiency of OPFRs in tap-water supply systems. The results showed that all water samples contained 9.25–224.74 ng·L^−1^ of OPFRs, predominantly tris(1-chloro-2-propyl) phosphate (TCPP), tris(2-chloroethyl) phosphate (TCEP), triphenyl phosphate (TPHP), and tributyl phosphate (TBP) [16]. Van der Veen et al. studied phosphorus flame retardants in air, water, sediment, and biota. They found 47 μg·m^−3^, 24 mg·kg^−1^, and 379 ng·L^−1^ of phosphorus flame retardants in air, sediments, and surface water, respectively. Some phosphorus flame retardants were severely carcinogenic, whereas TPHP and diphenylcresylphosphate were toxic to aquatic organisms [17,18]. Thus, the release and exposure of phosphorus in water should be controlled to ensure human health and environmental safety.

The potential harm of organophosphorus to the environment can be effectively avoided by introducing polyamide molecular chains through copolymerization of reactive flame retardants or blending macromolecular flame retardants with polyamide [19,20,21]. We reacted flame retardant with phosphaphenanthrene structure 9,10-dihydro-10-[2,3-di(hydroxycarbonyl)propyl]-10-phosphaphenanthrene-10-oxide (DDP) with decamethylene diamine to form a DDP salt; this salt was copolymerized with caprolactam to prepare intrinsic flame-retardant PA6. The flame-retardant PA6 containing 5 wt% DDP exhibited a limiting oxygen index (LOI) of 33.7% and a vertical burning rating of UL-94 V-0 [22]. In addition, we introduced flame retardant with phosphine oxide structure 2,3-dicarboxypropyl diphenyl phosphine oxide (DPDPO) as a reactive flame retardant into the PA6 molecular chain to obtain a copolymerized flame-retardant PA6. The flame-retardant PA6 with 5 wt% DPDPO passed the V-0 rating (UL 94) with a LOI value of 31.7%. The results indicate that DPDPO promotes the degradation of PA6 at high temperature, and mainly acts by the quenching effect of phosphorus-containing radicals in the gas phase [23]. Liang et al. synthesized macromolecular flame retardant (PFR) with phosphaphenanthrene structure based on DDP, decamethylene diamine, and caprolactam. The vertical combustion test of flame-retardant PA6 with 0.3 wt% phosphorus passed the V-0 grade (UL-94). The LOI of flame-retardant PA6 with 0.5 wt% phosphorus content was increased from 21.7% to 28.8% [24].

Similar to DDP with high flame-retardant efficiency, 2-carboxyethyl phenyl phosphinic acid (CEPPA) is an important reactive organophosphorus flame retardant with phosphine oxide structure that has been widely applied in improving the flame retardancy of polyamide. Chen et al. prepared flame-retardant copolyamide 66 (FR–PA66) successfully by in situ polymerization with CEPPA. The LOI values were increased from 24.0% to 28.0% by adding 6 wt% of CEPPA, and all FR–PA66 samples were rated as V-0 in UL-94 tests. The thermal stability analysis indicated that in situ polymerization with CEPPA effectively decreased the initial decomposition temperature and increased the amount of char residue [25]. Yang et al. synthesized flame-retardant PA56 copolymers (FRPA56s) with the introduction of CEPPA monomer. The crystallization temperature, melting temperature, and crystallinity were reduced, and the degradation pathway was changed with the introduction of CEPPA. The FRPA56 containing 7.8 mol% CEPPA passed the V-0 rating in the UL-94 vertical burning test, and its total heat release was reduced by 20% relative to that of PA56, reflecting the high flame-retardant efficiency of CEPPA [26]. Although the copolymerization method can effectively avoid the environmental harm of small-molecule precipitation, the copolymerization method has high requirements for equipment and processes, and the copolymerization method for polyamide has not been used commercially. Therefore, we propose the preparation of CEPPA to obtain macromolecular flame retardants and to obtain flame-retardant PA6 by blending macromolecular flame retardants with conventional PA6. In general, small-molecule flame-retardant CEPPA needs to be introduced into PA6 by a complex copolymerization method, and the macromolecular flame retardant based on CEPPA in this study can be introduced into PA6 by a simple melt blending method. Moreover, the macromolecular flame retardant has the advantage of not migrating and polluting the environment.

Herein, a macromolecular flame retardant (MFR) was synthesized via melt polycondensation using CEPPA, hexamethylene diamine (HMDA), and caprolactam (CPL) as raw materials. Then, flame-retardant PA6 (FR–PA6) was prepared by blending the MFR with PA6. The chemical structure, melting and crystallization properties, thermal stability, flame-retardant properties, and flame-retardant mechanism were subsequently studied.

## 2. Experimental Method

### 2.1. Materials

CEPPA (>99% purity) was purchased from Hubei Longxin Chemical Industry Co., Ltd. (Macheng, China). CPL (>99.9% purity) was obtained from Ube Industries, Ltd. (Tokyo, Japan). PA6 (relative viscosity 2.4, 2400A) was purchased from Hangzhou Juheshun New Materials Co., Ltd. (Hangzhou, China). HMDA (>99% purity), ethanol (EtOH; >99% purity), dimethyl sulfoxide (DMSO, >99% purity), *N*,*N*-Dimethylformamide (DMF, >99.5% purity), potassium bromide (>99% purity), hexafluoroisopropanol (HFIP, >99.5% purity), sodium trifluoroacetate (>99% purity), and trifluoroacetic acid-d (>99.5% purity) were purchased from Aladdin Chemistry Co., Ltd. (Shanghai, China). Deionized water was produced in the laboratory.

### 2.2. Preparation of Flame-Retardant Polyamide 6

**Scheme 1:**
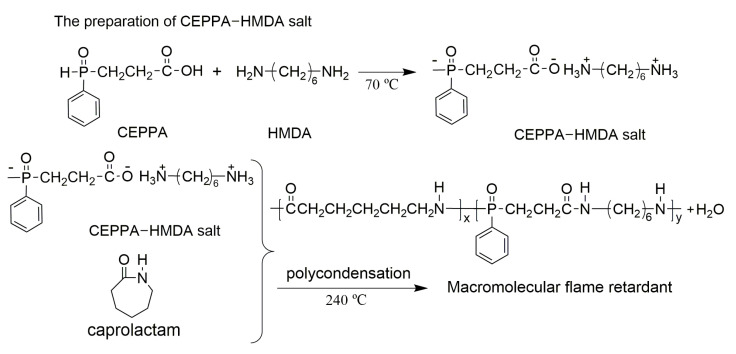
Synthesis process of MFR.

Scheme 1 shows the synthesis process for the MFR. First, 0.2 mol HMDA and 1000 mL ethanol were added to a round-bottom flask to obtain the HMDA solution at 50 °C. Then, 0.2 mol CEPPA was gradually added to it and heated to 70 °C for 5 h. The obtained CEPPA–HMDA salt solution was cooled down to room temperature (about 25 °C) and filtered with a Brinell funnel. The filter cake was washed with ethanol to remove unreacted monomers and dried in a vacuum oven at 60 °C for 8 h to obtain CEPPA–HMDA salt. Then, the synthesis of the MFR was carried out in a polymerization reactor. The CEPPA–HMDA salt, CPL, and deionized water were heated to 90 °C and stirred for 0.5 h. Next, the temperature of the reaction system was raised to 240 °C over a period of one hour, while the pressure in the reactor was increased to 2.0 MPa with the increase of temperature, and the temperature and pressure were maintained for 2 h for the pre-polymerization of the MFR. Then, the pressure of the polymerizer was reduced to 0 MPa within 1 h, and the final polycondensation of the MFR was carried out by vacuuming to −0.1 MPa for 3 h. The as-prepared MFR was then extracted with 100 °C water to remove the residual caprolactam and obtain purified MFR. As shown in Table 1, the MFR with CEPPA–HMDA salt contents of 10%, 20%, 30%, and 40% was labeled MFR-10, MFR-20, MFR-30, and MFR-40, respectively.

The MFR was dried with PA6 at 90 °C for 12 h and then blended with a screw extruder to obtain flame-retardant PA6 (FR–PA6). The FR–PA6 containing 0.3, 0.4, and 0.5 wt% of phosphorus was labeled FR–PA6-1, FR–PA6-2, and FR–PA6-3, respectively (Table 2).

### 2.3. Characterization

Fourier-transform infrared (FT–IR) spectroscopy was performed using a Niciletis 20 FT–IR spectrometer (Thermo Fisher Scientific, Waltham, MA, USA). CEPPA and CEPPA–HMDA were mixed with KBr and pressed into transparent sheets, and the MFR was measured using an attenuated total reflection (ATR) cell and a diamond crystal.

Nuclear magnetic resonance (^1^H-NMR and ^31^P-NMR) was performed using a Bruker Avance III NMR spectrometer (600 MHz) (Billerica, MA, USA). Trifluoroacetic acid-d was used as the solvent for the MFR.

Advanced polymer chromatography (APC) was performed using an instrument equipped with an Acquity chromatograph (Waters Corporation, Milford, MA, USA), a Dawn Heleos II evaporating light-scattering detector (Wyatt Technology Corporation, Santa Barbara, CA, USA), and an Optilab T-rEX differential refractive index detector (Wyatt Technology Corporation, Santa Barbara, CA, USA).

Thermogravimetric analysis (TGA) was conducted on an STA 2500 Regulus thermogravimetric analyzer (The NETZSCH Group, Selb, Germany) under nitrogen and air atmospheres at a heating rate of 10 °C·min^−1^.

Differential scanning calorimetry (DSC) analysis was conducted on a DSC 3 instrument (The Mettler Toledo Group, Zurich, Switzerland). The samples were first heated from room temperature to 250 °C at a heating rate of 40 °C·min^−1^ under a nitrogen atmosphere; this condition was maintained for 5 min to eliminate any previous thermal history. The sample was subsequently cooled down to 100 °C and reheated to 250 °C at a rate of 10 °C·min^−1^.

The crystallization behavior of the samples was observed using an Empyrean X-ray diffraction (XRD) (Malvern Panalytical, Malvern, UK) X’Celerator detector in the 2θ range from 15° to 35° at a scanning rate of 2°·min^−1^.

A tensile test was performed on an Instron 365 material testing machine (Norwood, MA, USA) according to ISO 527-1:2019 [27].

A vertical burning test (UL-94) was performed according to ASTM D3801 [28] using the F241 vertical combustion apparatus (Shanghai Qianshi Precision Electromechanical Technology Co., LTD, Shanghai, China) on a sample with dimensions of 127 × 12.7 × 3.2 mm^3^.

LOI was measured according to ASTM D2863 [29] using the F101 oxygen indexer (Nanjing Jionglei instrument equipment Co., LTD, Nanjing, China). The dimensions of testing specimens were 100 × 10 × 4 mm^3^. Scanning electron microscopy (SEM) images were acquired on a Zeiss Sigma 500 field-emission scanning electron microscope (Oberkochen, Germany).

A cone calorimetric test was carried out on a VOUCH 6810 cone calorimeter (Suzhou Yangyi Vouch Testing Technology Co., LTD, Suzhou, China) according to ISO 5660 [30] with a sample of 100 × 100 × 3 mm^3^ under a heat flow of 35 kW·m^−2^.

Pyrolysis–gas chromatography–mass spectrometry (Py–GC–MS) was performed using a PY-3030D spectrometer (Shimadzu Corporation, Kyoto, Japan) at a pyrolysis temperature of 600 °C.

## 3. Results and Discussion

### 3.1. Properties of the MFR

#### 3.1.1. Chemical Structural of the MFR

**Figure 1:**
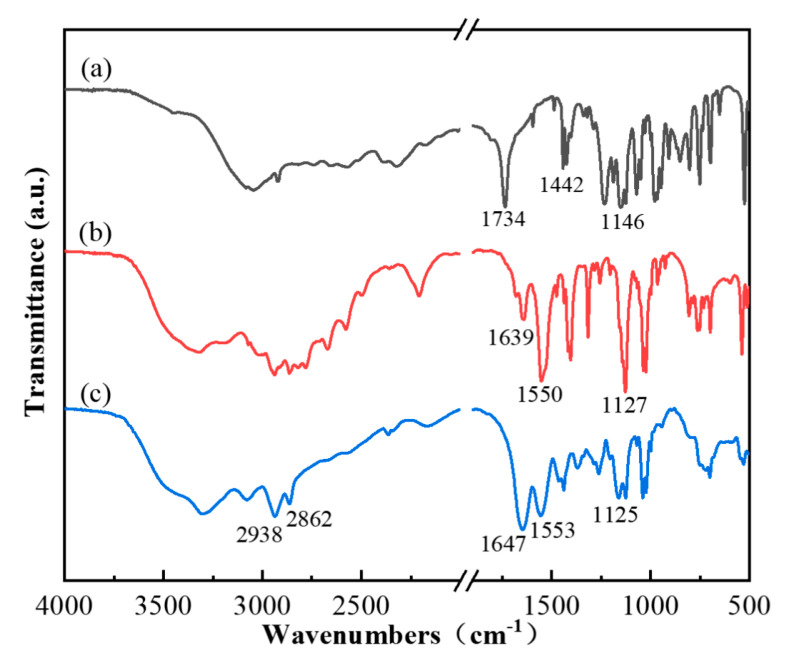
FT–IR spectra of CEPPA (**a**), CEPPA–HMDA salt (**b**), and MFR (**c**).

The FT–IR spectra of CEPPA, CEPPA–HMDA salt, and the MFR were obtained to analyze the structure of the MFR (Figure 1). The spectrum for CEPPA (Figure 1a) shows an absorption peak at 1734 cm^−1^ corresponding to the carbonyl stretching vibration of carboxylic acids and absorption peaks at 1442 and 1146 cm^−1^ corresponding to the P–C and P=O stretching vibrations of CEPPA. The amino group, an electron-absorbing group, caused the shift of the FT–IR characteristic peak of C=O. As shown in Figure 1c, the absorption peaks at 2938 and 2862 cm^−1^ are caused by the –CH_2_ stretching vibrations of HMDA [22]. The absorption peak at 1125 cm^−1^ corresponds to the P=O stretching vibration of CEPPA. CEPPA’s carboxyl absorption peak at 1734 cm^−1^ disappeared, and new peaks appeared at 1647 cm^−1^ (C=O stretching vibration of the amide bond) and 1553 cm^−1^ (N–H deformation and C–N stretching of the amide bond) [23,31], which preliminarily proved the successful synthesis of the MFR.

**Figure 2:**
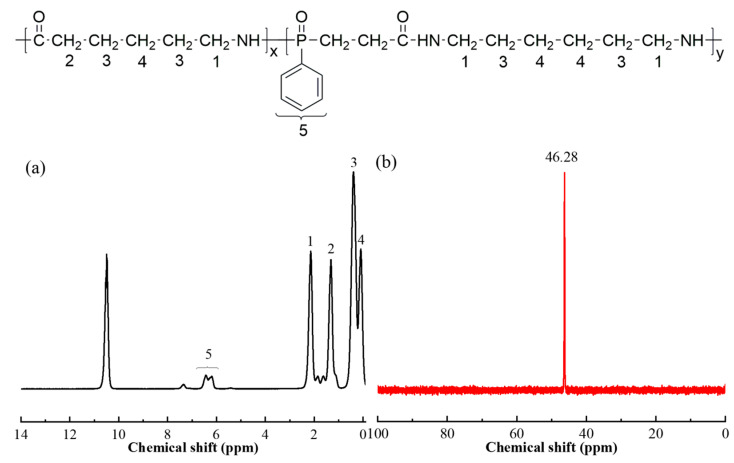
^1^H-NMR (**a**) and ^31^P-NMR (**b**) spectra of the MFR.

The MFR structure was further validated based on the ^1^H-NMR and ^31^P-NMR spectra (Figure 2). In the ^1^H-NMR spectrum (Figure 2a), the chemical shifts at 2.14 (1), 1.31 (2), 0.68 (3), and 0.09 ppm (4) corresponded to hydrogens on the MFR. The peaks at 6.03–6.68 ppm (5) accounted for the chemical shifts of hydrogens on the aromatic ring. Figure 2b shows the ^31^P-NMR spectra of the MFR, where the absorption peak at 46.28 ppm can be attributed to the phosphorus atom signals of the MFR. Thus, the FT–IR, ^1^H-NMR, and ^31^P-NMR results confirmed successful preparation of the MFR.

#### 3.1.2. Molecular Weight and Molecular Weight Distribution of the MFR

**Figure 3:**
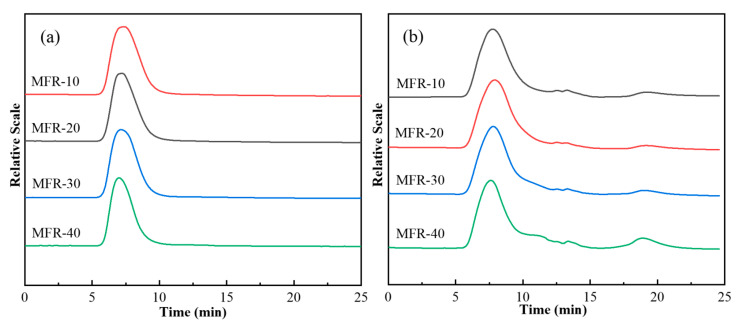
ELSD (**a**) and RID (**b**) spectra of the MFR.

APC was performed to determine the molecular weight and molecular weight distribution of the MFR. Figure 3 shows the spectra of the MFR obtained using the evaporative light-scattering detector (ELSD) and refractive index detector (RID). The concentration gradient curve of dn/dc and refractive index standard curve of MFR are shown in Figure S1. The molecular weights are listed in Table 3, where *M_n_*, *M_w_*, and PDI denote the number average molecular weight, weight average molecular weight, and polydispersity index, respectively. As the CEPPA–HMDA salt content increased, the molecular weight gradually decreased from 24,700 to 15,300 g·mol^−1^, and the molecular weight distribution showed a gradually wider trend. This was because of the steric hindrance effect of the aromatic ring on CEPPA that prevented further polymerization of the MFR [32,33]. In addition, the solubility test shows that MFR with different molecular weights all exhibit significantly better solvent resistance compared to CEPPA (Table S1).

#### 3.1.3. Thermal Properties of the MFR

**Figure 4:**
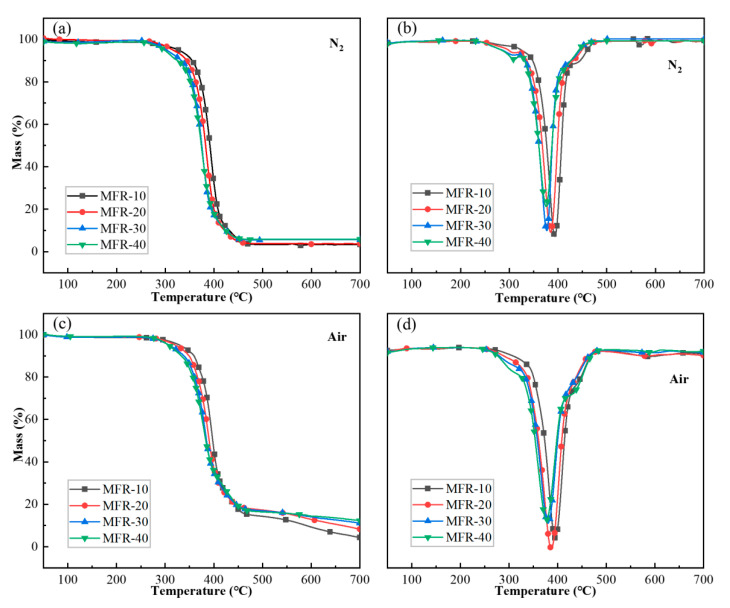
TG and DTG curves of the MFR. (**a**) TG under N_2_; (**b**) DTG under N_2_; (**c**) TG under Air; (**d**) DTG under Air.

TGA was performed to assess the thermal stability of the MFR. Figure 4 shows the TG and DTG curves under nitrogen and air atmospheres, and their corresponding data are listed in Table 4. *T*_5%_ and *T_max_* denoted the initial decomposition and maximum weight-loss temperatures, respectively. With the increase of the CEPPA content, the *T*_5%_ and *T_max_* values of the MFR decreased under nitrogen and air atmospheres. The P–C bond energy of CEPPA was 260 kJ mol^−1^, lower than the C–C bond of the polyamide chain segment. The breakage of the P–C bond reduced the thermal stability of the MFR [34]. The carbon residue amounts increased with increasing MFR content under nitrogen and air atmospheres. The TGA results indicate that the MFR has high thermal stability and can meet the temperature requirements of injection molding and melt spinning of PA6.

**Figure 5:**
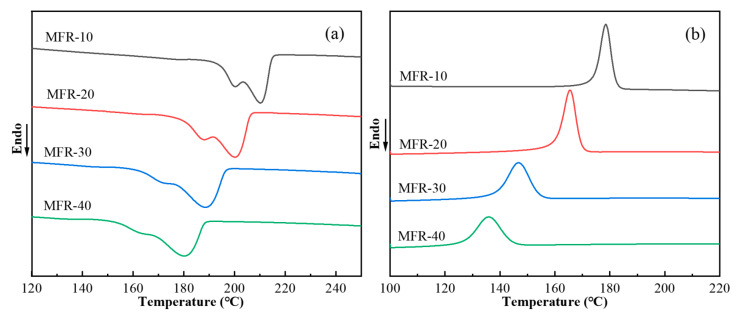
DSC heating curves (**a**) and cooling curves (**b**) of the MFR.

Melt and crystallization behavior can be used to analyze and evaluate the processability of flame retardants. The melt crystallization behavior of the MFR was analyzed by DSC. Figure 5 shows the DSC heating and cooling curves of the MFR, and Table 5 summarizes the corresponding DSC data. The DSC heating curves of CEPPA and CEPPA–HMDA salt is shown in Figure S2. As the CEPPA–HMDA salt content increased, the melting and crystallization peaks of the MFR shifted to lower temperatures. The *T_m_* and *T_c_* of the MFR decreased from 211 °C and 179 °C for MFR-10 to 180 °C and 136 °C for MFR-40, respectively. When the CEPPA–HMDA content increased to ≥30 wt%, the MFR showed a certain stickiness, which easily blocked the feed port of the twin screw; this was not conducive to subsequent processing. This phenomenon was observed because the aromatic ring structure of CEPPA destroyed the MFR main chain regularity [35]. Therefore, the blending of MFR-20 and PA6 was selected to prepare FR–PA6 because of its high phosphorus content and best melting processing performance.

### 3.2. Properties of FR–PA6

#### 3.2.1. Thermal Properties of FR–PA6

**Figure 6:**
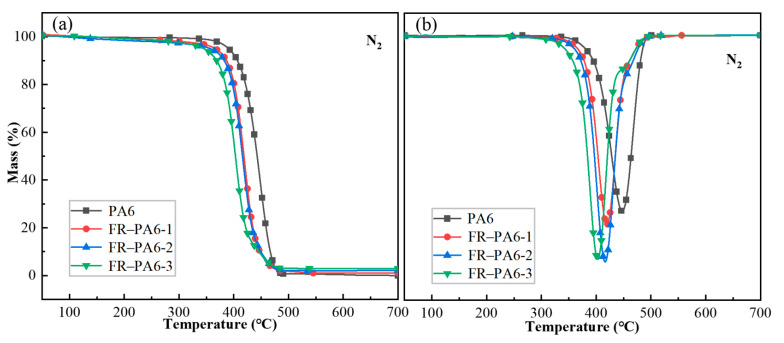
TG (**a**) and DTG (**b**) curves of PA6 and FR–PA6.

The degradation behavior and char formation properties of PA6 and FR–PA6 were effectively studied via thermogravimetric analysis. Figure 6 shows the TG and DTG curves of PA6 and FR–PA6 under nitrogen atmospheres. Table 6 shows the corresponding data in detail. The *T*_5%_ and *T_max_* of PA6 were 386.5 °C and 469.2 °C, respectively. The *T*_5%_ and *T_max_* of FR–PA6 were lower than those of PA6. As the MFR content increased, the *T*_5%_ and *T_max_* of FR–PA6 gradually decreased from 367.7 °C and 464.8 °C to 340.1 °C and 460.9 °C, respectively. The initial decomposition temperature of FR–PA6 was still above 340 °C, indicating that it has good thermal stability and meets the requirements of processing and use. However, the carbon residue rate gradually increased from 0.7% to 2.3%. It can be seen that the introduction of MFR does not help PA6 form a dense enough carbon layer to isolate heat and oxygen. On the contrary, the addition of MFR reduces the decomposition temperature of PA6. The earlier decomposition temperature means that the molecular chains break at the earlier flame temperature, which promotes faster formation of melt droplets during combustion, taking heat away from the burning PA6 timely [36].

**Figure 7:**
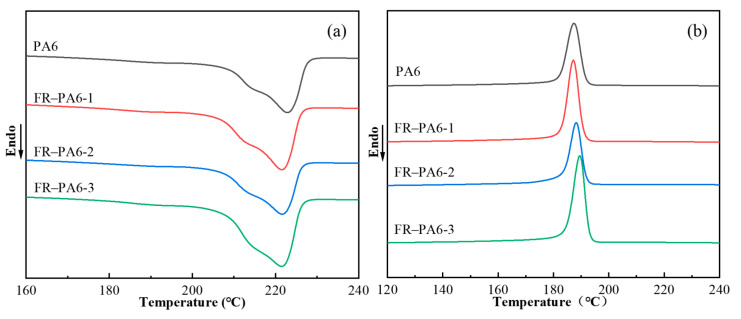
DSC heating (**a**) and cooling (**b**) curves of PA6 and FR–PA6.

**Figure 8:**
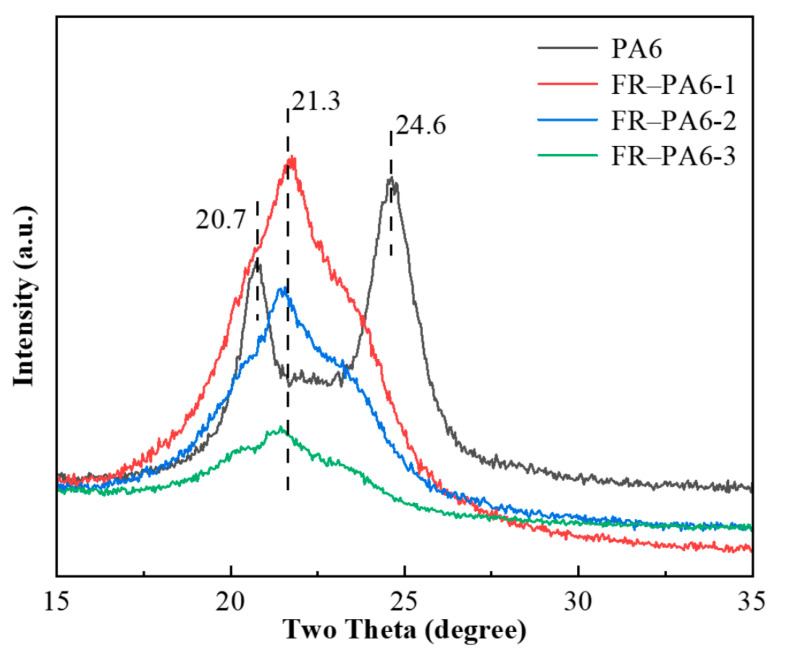
XRD patterns of PA6 and FR–PA6.

The thermal transition and crystallinity of PA6 and FR–PA6 were studied via DSC. Figure 7 shows the DSC heating and cooling curves of PA6 and FR–PA6, and the corresponding results are listed in Table 7. The *T_m_* and *T_c_* of PA6 were 223 °C and 187 °C, respectively. The *T_m_* and *T_c_* of MFR-20 were 200 °C and 166 °C, respectively. As the content of MFR-20 increased, the *T_m_* of FR–PA6 decreased from 223 °C to 221 °C and its *T_c_* increased from 187 °C to 189 °C. The aromatic ring structure in MFR destroys the regularity of the PA6 molecular chain, resulting in a lower melting temperature. However, there may be heterophase nucleation, which promotes the crystallization of PA6 at higher temperatures. In general, the introduction of MFR has little impact on the melting temperature and crystallization temperature of FR–PA6. Figure 8 shows the X-ray diffraction patterns of PA6 and flame-retardant PA6. PA6 is a crystalline polymer with two distinct crystal structures, the α and γ crystals. The characteristic diffraction peaks of the α crystal are 2θ of 20.7° and 24.6°, while the γ crystal corresponds to 2θ of 21.3°. It can be seen that pure PA6 is a stable α crystal, and flame-retardant PA6 has a strong γ crystal diffraction peak. This is because the introduction of MFR obstructs the movement of the molecular chains, and the movement of the chain segments of PA6 molecules tends to form γ crystals when hindered.

#### 3.2.2. Mechanical Properties of FR–PA6

The mechanical properties of PA6 and FR–PA6 were evaluated via tensile tests, the results are summarized in Table 8 and the stress–strain curves are shown in Figure S3. Compared with those of pure PA6, the tensile strength and elongation at break of FR–PA6 decreased upon adding MFR. Moreover, the mechanical properties degraded with increasing MFR content. In the literature, Li et al. added 14 wt% aluminum diethylphosphinate to PA6; the vertical combustion test of the material reached the UL94 V-0 class, but the tensile strength decreased by about 25% [37]. Zhang et al. synthesized intrinsic flame-retardant PA6 by introducing reactive flame-retardant DDP into the synthesis process of PA6. The tensile strength of the intrinsic flame retardant PA6 decreased from 76.92 MPa to 43.48 MPa (a decrease of 43.5%), and the modulus decreased from 1996.43 MPa to 1895.6 MPa [38]. The introduction of a large number of flame retardants usually leads to a decline in the mechanical properties of materials. In this study, the tensile strength of PA6 was 63.2 MPa, whereas that of FR–PA6-3 was 46.2 MPa (26.9% lower than PA6). The elongation at break of PA6 was 26.5%, whereas that of FR–PA6 decreased significantly. In addition, the Young’s modulus of FR–PA6 is about 20% lower than that of PA6, which indicates that the introduction of MFR reduces the deformation resistance of PA6. This is mainly because the introduction of MFR with a benzene ring structure reduces the hydrogen bond density and increases the defect weakness of FR–PA6, thus reducing the mechanical properties of FR–PA6. In addition, the molecular weight of MFR is relatively low, and the synthesis of MFR with higher molecular weight may be helpful to improve the mechanical properties of FR–PA6.

#### 3.2.3. Flame-Retardant Property and Mechanism

**Figure 9:**
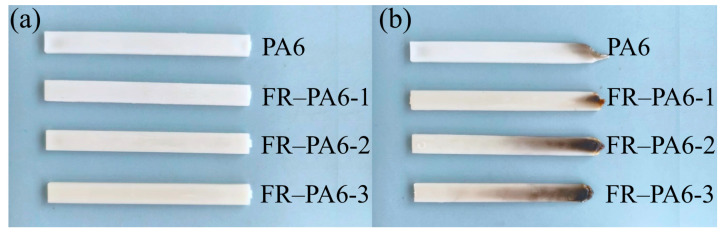
Before (**a**) and after (**b**) vertical combustion tests for PA6 and FR–PA6.

The flammability of materials can be ranked based on the vertical combustion test and LOI test. Figure 9 shows the changes to PA6 and FR–PA6 before and after the vertical burning test. Table 9 shows the test results of the vertical combustion test and limiting oxygen index test for PA6 and FR–PA6. In the vertical combustion test of PA6, burns with severe melt dripped during combustion. The cotton was ignited by the molten droplets, indicating that PA6 achieved a V-2 rating according to the UL-94 criterion. Upon adding the MFR, FR–PA6 samples were more likely to form melt droplets that carried away the burning heat timely. During the test, the heat caused by the external flame was taken away by the melt droplets timely; when the external flame was removed from the test, the burning FR–PA6 lost its heat source and quickly stopped burning. Thus, the total number of melt droplets from FR–PA6 was reduced, and the self-extinguishing time of the droplets was significantly reduced, so a small amount of non-flame melt droplets did not ignite the cotton. FR–PA6 achieved a V-0 rating according to the UL-94 criterion. The LOI obviously improved upon adding the MFR. Compared with that of PA6, the LOI of FR–PA6-3 increased from 21.8% to 28.2%. It can be seen that the self-extinguishing time of the fire is greatly shortened, and the flameless melt drops take away the heat timely, which jointly improves the flame-retardant performance of FR–PA6.

**Figure 10:**
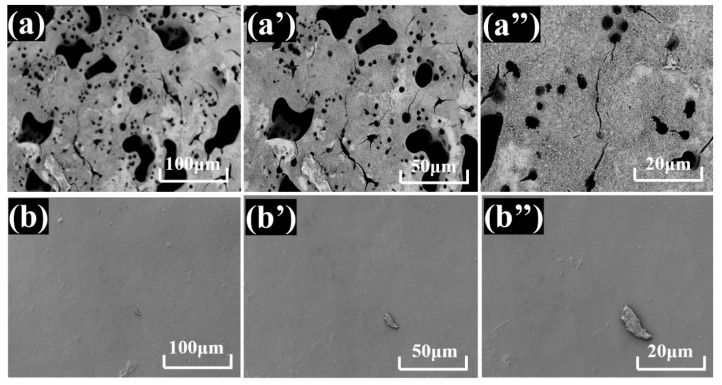
SEM images of the residues in PA6 (**a**, **a′** and **a″**) and FR–PA6-3 (**b**, **b′** and **b″**).

The effect of residue formation on the flame retardancy of FR–PA6-3 was studied by analyzing the carbon residue via SEM. Figure 10 shows SEM images of PA6 and FR–PA6-3 surfaces. The surface of the carbon layer on PA6 is loose and porous, whereas that of FR–PA6-3 is relatively compact and flat. The introduction of MFR is conducive to improving the carbon forming performance of FR–PA6. However, combined with the TGA results, it can be seen that the mass of carbon residue is small, and the carbon layer formed is thin, which cannot play a role in isolating heat and oxygen. Moreover, the phosphorus content in the carbon layer is only 3.23%, indicating that MFR primarily exerts its flame-retardant effect by trapping burning free radicals in the gas phase through phosphorus-containing free radicals, rather than promoting the formation of carbon layer in the condensed phase (Figure S4).

**Figure 11:**
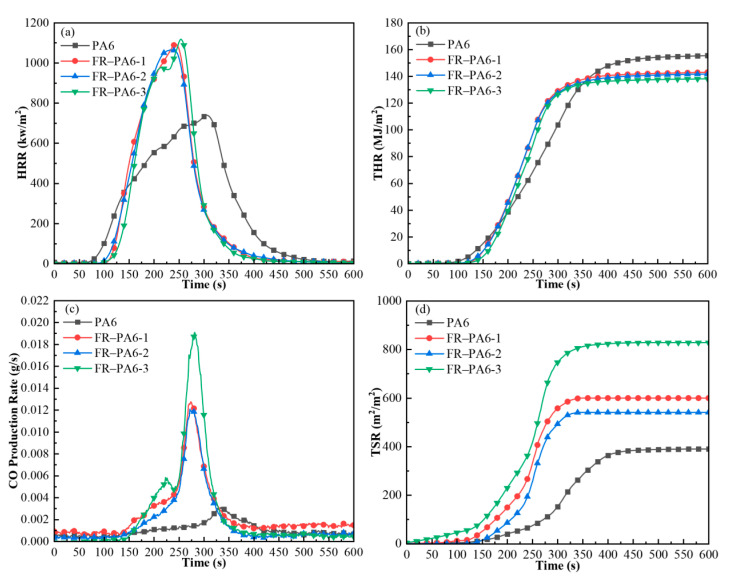
HRR (**a**), THR (**b**), CO production rate (**c**), and TSR (**d**) curves of samples.

The combustion behaviors and flame-retardant mechanisms of materials can be deduced via cone calorimetry. The HRR curves, total heat release (THR) curves, CO production rate, and total smoke release (TSR) curves of PA6, FR–PA6-1, FR–PA6-2, and FR–PA6-3 are shown in Figure 11, and various combustion parameters are listed in Table 10. Compared with pure PA6, the p-HRR values of FR–PA6-3 increased from 753 to 1197 kW·m^−2^, and THR decreased from 158 to 138 MJ·m^−2^, respectively. It was observed that the FR–PA6 samples exhibited a substantial and unexpected increase in p-HRR, which is inconsistent with the results of the vertical burning (UL-94) test. Mechanism analysis revealed that the melt flow behavior induced by the degradation of the MFR promoted heat dissipation through the formation of melt droplets during the vertical burning test. However, under radiative heating in the cone calorimetry test, the degradation of the MFR led to accelerated heat accumulation over a short duration. The heat generated by rapid decomposition could not be removed in time, ultimately leading to an elevated p-HRR. Additionally, the limited reduction in THR further supports the conclusion that the flame-retardant mechanism of MFR relies less on char layer formation. The MFR failed to promote the formation of a dense char layer capable of providing effective thermal insulation. Notably, the av-EHC of FR–PA6-3 was reduced by 32.2% compared to PA6, and the SEA of FR–PA6-3 was improved by 66.7% compared to PA6. The EHC denotes the heat released by volatilization in a meteorological flame. The SEA represents the amount of smoke released during combustion per unit mass of materials. The lower av-EHC values and higher SEA show that the gas-phase combustion is not complete, indicating a gas-phase flame-retardant mechanism of the MFR in PA6 [39,40,41]. Figure 11c,d show that FR–PA6-3 produced higher amounts of CO and smoke than PA6 during combustion. The TSR value increased from 390 m^2^·m^−2^ for PA6 to 827 m^2^·m^−2^ for FR–PA6-3. The increased CO and TSR values indicate incomplete combustion, further confirming that the MFR was a flame retardant that acts in the gas phase [42,43,44]. The MFR produced phosphorus-containing free radicals, which can capture active free radicals (H· and OH·) and end the chain reaction of combustion. The char residue of FR–PA6-3 was only 0.54%. Consistent with the TGA results, the amount of char residue could not form an effective barrier or prevent the heat transfer and diffusion of combustible gases in the condensed phase.

**Figure 12:**
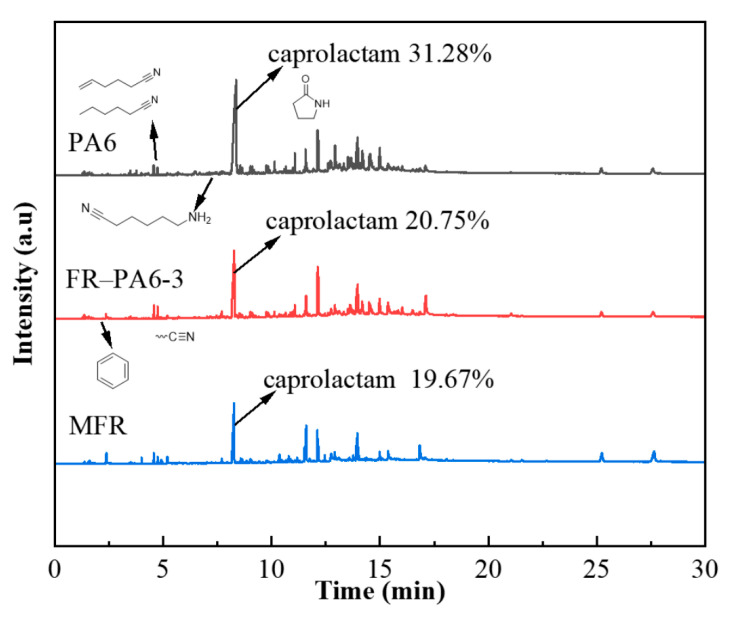
Py–GC–MS spectra of PA6, FR–PA6-3, and MFR.

The samples were subjected to Py–GC–MS to further analyze the flame-retardant mechanism of the MFR in the gas phase. Figure 12 shows the pyrolysis spectra of the PA6, FR–PA6-3, and MFR. The characteristic peaks of PA6 mainly included caprolactam, hexanenitrile, carbon dioxide, cyclopentanone, 5-cyano-1-pentene, and 6-aminoisocyanide. The characteristic peaks of the MFR were similar to those of FR–PA6-3 but with different absorption intensities. Except for the main pyrolysis products of PA6, benzene rings were found in the MFR and FR–PA-3 at 4.83 and 5.01 min of pyrolysis. In addition, many unsaturated long-chain terminal nitriles were found after ~4 min of pyrolysis due to amide bond breakage. It can be seen from the Py–GC–MS data of the MFR that the P-C bond of the CEPPA chain segment in MFR will break earlier than the polycaprolactam chain segment, which will affect the molecular chain breaking process of polycaprolactam and reduce the concentration of caprolactam and other flammable gases generated during degradation. Compared with PA6, the related area of caprolactam produced during FR–PA6-3 cleavage decreased from 31.28% to 19.67% (a decrease of 37.1%). The flammability of PA6 is related to the combustible caprolactam produced by pyrolysis at high temperature. The less caprolactam produced, the harder PA6 is to burn. Combined with the cone calorimetry results, it can be seen that MFR decomposition in the gas phase releases phosphorus-containing free radicals to interrupt the combustion chain reaction and reduce the concentration of the combustible caprolactam. In the condensed phase, the MFR promotes faster formation of melt droplets during combustion, taking heat away from the burning PA6 timely. Therefore, the above two mechanisms of MFR work together to play a flame-retardant role in PA6.

## 4. Conclusions

Herein, an MFR with a phosphine oxide structure was synthesized via the copolymerization of CEPPA, HMDA, and CPL. The addition of the as-synthesized MFR improved the flame retardancy of PA6. FR–PA6 with 0.3 wt% of phosphorus passed the V-0 rating, and the LOI of FR–PA6 with 0.5 wt% of phosphorus increased from 21.8% to 28.2%. Compared with pure PA6, the av-EHC of FR–PA6-3 was reduced by 32.2% and the SEA was increased by 66.7%. The flame-retardant mechanism of the MFR mainly comprised the quenching effect of phosphorus-containing free radicals and reducing the concentration of the combustible caprolactam in the gas phase and promoting the timely drop of melt droplets to carry away heat in the condensed phase. At present, the molecular weight of the prepared MFR is not high enough, which has an influence on the mechanical properties of PA6. In the future, the molecular weight of MFR can be further improved via molecular structure design and improvement of the polymerization method.

## Figures and Tables

**Table 1:** Formulations for the synthesis of MFR.

Sample	CEPPA–HMDA Salt (g)	CPL (g)	H_2_O (g)	Ratio of CEPPA–HMDA Salt (%)
MFR-10	120	1080	130	10
MFR-20	240	960	130	20
MFR-30	360	840	130	30
MFR-40	480	720	130	40

**Table 2:** Formulations for the preparation of FR–PA6.

Sample	MFR-20 (wt%)	PA6 (wt%)	Theoretical P (ppm)
FR–PA6-1	18.8	100	3000
FR–PA6-2	26.7	100	4000
FR–PA6-3	35.7	100	5000

**Table 3:** APC results of the MFR.

Sample	*M_n_* (g·mol^−1^)	*M_w_* (g·mol^−1^)	PDI
MFR-10	2.47 × 10^4^	3.38 × 10^4^	1.37
MFR-20	1.95 × 10^4^	3.00 × 10^4^	1.54
MFR-30	1.56 × 10^4^	2.51 × 10^4^	1.61
MFR-40	1.53 × 10^4^	2.40 × 10^4^	1.57

**Table 4:** Thermogravimetry results of the MFR.

Sample	N_2_ Atmosphere			Air Atmosphere		
	*T*_5%_ (°C)	*T_max_* (°C)	Char (%)	*T*_5%_ (°C)	*T_max_* (°C)	Char (%)
MFR-10	327.0	453.6	3.3	330.4	458.5	4.3
MFR-20	309.0	441.2	4.0	312.4	445.1	5.2
MFR-30	308.2	439.1	6.4	308.5	440.3	6.1
MFR-40	302.9	433.0	6.6	304.4	439.9	6.9

**Table 5:** DSC results of the MFR.

Sample	*T_c_* (°C)	*T_m_* (°C)	∆H_m_ (J·g^−1^)
MFR-10	179	211	32
MFR-20	166	200	33
MFR-30	147	189	27
MFR-40	136	180	35

**Table 6:** TGA results of PA6 and FR–PA6.

Sample	*T*_5%_ (°C)	*T_max_* (°C)	Char (%)
PA6	386.5	469.2	0.7
FR–PA6-1	367.7	464.8	1.8
FR–PA6-2	358.6	463.3	2.2
FR–PA6-3	340.1	460.9	2.3

**Table 7:** DSC results of PA6 and FR–PA6.

Sample	*T_c_* (°C)	*T_m_* (°C)	∆H_m_ (J·g^−1^)	X_c_ (%)
PA6	187	223	50	22
FR–PA6-1	187	222	50	22
FR–PA6-2	188	222	49	21
FR–PA6-3	189	221	70	31

**Table 8:** Mechanical properties of PA6 and FR–PA6.

Sample	Tensile Strength (MPa)	Elongation at Break (%)	Young’s Moduli (MPa)
PA6	63.2 ± 3.5	26.5 ± 4.2	1968.8 ± 203.8
FR–PA6-1	57.9 ± 3.4	3.7 ± 1.3	1564.9 ± 338.9
FR–PA6-2	49.5 ± 2.4	3.2 ± 1.1	1546.9 ± 339.9
FR–PA6-3	46.2 ± 3.2	3.0 ± 1.2	1540.0 ± 363.8

**Table 9:** Vertical combustion test results and LOI values of samples.

Sample	Vertical Combustion Test		LOI Value (%)
	Cotton Ignited or Not	Rating	
PA6	Yes	V-2	21.8 ± 0.1
FR–PA6-1	No	V-0	25.7 ± 0.1
FR–PA6-2	No	V-0	27.2 ± 0.1
FR–PA6-3	No	V-0	28.2 ± 0.1

**Table 10:** Cone calorimetric test results of the samples.

Sample	p-HRR (kw/m^2^)	THR (MJ/m^2^)	av-EHC (MJ/kg)	SEA (m^2^/kg)	av-CO (kg/kg)	TSR (m^2^/m^2^)	Char (%)
PA6	753	158	25.8	84	0.0280	390	0.01
FR–PA6-1	1116	144	20.0	84	0.0597	599	0.33
FR–PA6-2	1098	142	19.1	78	0.0404	541	0.50
FR–PA6-3	1197	138	17.5	140	0.0528	827	0.54

## Data Availability

The original contributions presented in the study are included in the article, further inquiries can be directed to the corresponding author.

## Supplementary Materials

The following supporting information can be downloaded at: https://www.mdpi.com/article/10.3390/polym17040475/s1, Figure S1: Concentration gradient curve of dn/dc (a) and refractive index standard curve of MFR (b); Figure S2: DSC heating curves of CEPPA and CEPPA–HMDA salt; Figure S3: Stress–strain curves of PA6 and FR–PA6; Figure S4: SEM-EDS images of PA6 (a) and FR–PA6 (b) carbon layer; Table S1: Solubility properties of CEPPA and MFR in different solvents (25 °C).

## References

[1] Faramarzi I., Razzaghi-Kashani M. (2015). Improvements in tribological properties of polyamide 6 by application of aramid pulp. Iran. Polym. J..

[2] Crespo J., Parres F., Peydró M., Navarro R. (2012). Study of rheological, thermal, and mechanical behavior of reprocessed polyamide 6. Polym. Eng. Sci..

[3] Zheng T., Wang W., Liu Y. (2021). A novel phosphorus-nitrogen flame retardant for improving the flame retardancy of polyamide 6: Preparation, properties, and flame retardancy mechanism. Polym. Adv. Technol..

[4] Guo X., Liu L., Feng H., Li D., Xia Z., Yang R. (2023). Flame Retardancy of Nylon 6 Fibers: A Review. Polymers.

[5] Li H., Chen L., Li Z., Wang W., Liu Y., Huang X., Liu Y. (2023). Synergistic effect of SiO2 doped g-C3N4 and ammonium polyphosphate on flame retardancy of PA6 composites. J. Polym. Res..

[6] Wang L., Zhang L., Fischer A., Zhong Y., Drummer D., Wu W. (2018). Enhanced thermal conductivity and flame retardancy of polyamide 6/flame retardant composites with hexagonal boron nitride. J. Polym. Eng..

[7] Vasiljević J., Čolović M., Jerman I., Simončič B. (2018). Recent Advances in Production of Flame Retardant Polyamide 6 Filament Yarns. Tekstilec.

[8] Adner D., Helmy M., Otto T., Schellenberg J., Schadewald A. (2018). A macromolecular halogen-free flame retardant and its effect on the properties of thermoplastic polyesters. Fire Mater..

[9] Yurddaskal M., Celik E. (2018). Effect of halogen-free nanoparticles on the mechanical, structural, thermal and flame retardant properties of polymer matrix composite. Compos. Struct..

[10] Huo S., Song P., Yu B., Ran S., Chevali V.S., Liu L., Fang Z., Wang H. (2021). Phosphorus-containing flame retardant epoxy thermosets: Recent advances and future perspectives. Prog. Polym. Sci..

[11] Velencoso M.M., Battig A., Markwart J.C., Schartel B., Wurm F.R. (2018). Molecular Firefighting—How Modern Phosphorus Chemistry Can Help Solve the Challenge of Flame Retardancy. Angew. Chem. Int. Ed. Engl..

[12] Carbone S., Drigo N., Huang K., Lehner S., Jovic M., Bifulco A., Gooneie A., Aronne A., Gaan S. (2024). Developing flame retardant solutions for partially aromatic polyamide with phosphine oxides. Mater. Des..

[13] Schartel B. (2010). Phosphorus-based Flame Retardancy Mechanisms—Old Hat or a Starting Point for Future Development?. Materials.

[14] Xiang H., Li L., Chen W., Yu S., Sun B., Zhu M. (2017). Flame retardancy of polyamide 6 hybrid fibers: Combined effects of α -zirconium phosphate and ammonium sulfamate. Prog. Nat. Sci..

[15] Huang B., Ma M., Liu Z., Jiang Z., Chen S., Shi Y., He H., Zhu Y., Wang X. (2024). A strategy toward improving flame retardancy and thermal oxidative stability of polyamide 6 based on cuprous diethylphosphinate. Polymer.

[16] Zhang Q., Li J., Lin S., Ying Z., Hu S., Wang Y., Mo X. (2022). Organophosphate flame retardants in Hangzhou tap water system: Occurrence, distribution, and exposure risk assessment. Sci. Total. Environ..

[17] van der Veen I., de Boer J. (2012). Phosphorus flame retardants: Properties, production, environmental occurrence, toxicity and analysis. Chemosphere.

[18] Zhou R., Lin L., Zeng B., Yi X., Huang C., Du K., Liu X., Xu Y., Yuan C., Dai L. (2022). Diblock Copolymers Containing Titanium-Hybridized Polyhedral Oligomeric Silsesquioxane Used as a Macromolecular Flame Retardant for Epoxy Resin. Polymers.

[19] Parcheta-Szwindowska P., Habaj J., Krzemińska I., Datta J. (2024). A Comprehensive Review of Reactive Flame Retardants for Polyurethane Materials: Current Development and Future Opportunities in an Environmentally Friendly Direction. Int. J. Mol. Sci..

[20] Chen Z.-X., Zhao Z.-Y., Lu P., Xiao X.-X., He S., Deng C., Wang Y.-Z. (2023). Novel macromolecular flame retardant derived from sulfonated naphthalene monomer for simultaneous fire safety and high performance of polycarbonate. Polym. Degrad. Stab..

[21] Sun Y., Pei X., Wang Z., Wu D., Wang X., Yu J., Yuan R., Li F. (2024). A macromolecular flame retardant for polyamide 6 and its filaments with enhanced fire safety, tensile and UV-blocking performance. Compos. Part B Eng..

[22] Liu K., Li Y., Tao L., Xiao R. (2018). Preparation and characterization of polyamide 6 fibre based on a phosphorus-containing flame retardant. RSC Adv..

[23] Liu K., Li Y., Tao L., Liu C., Xiao R. (2019). Synthesis and characterization of inherently flame retardant polyamide 6 based on a phosphine oxide derivative, *Polym. Degrad*. Stab.

[24] Liang B., Liu K., Dai J., Chen W., Lu W. (2024). Polymer-type flame retardants based on a DOPO derivative for improving the flame retardancy of polyamide 6: Preparation, properties and flame retardancy mode of action. Polym. Degrad. Stab..

[25] Chen X., Xu D., Zhang H., Feng X., Deng J., Pan K. (2019). In situ polymerization of flame retardant modification polyamide 6,6 with 2-carboxy ethyl (phenyl) phosphinic acid. J. Appl. Polym. Sci..

[26] Yang T., Gao Y., Liu X., Wang X., Ma B., He Y. (2022). Flame-retardant polyamide 56 with high fire safety and good thermal performance. Polym. Adv. Technol..

[27] (2019). Plastics—Determination of Tensile Properties.

[28] (2020). Standard Test Method for Measuring the Comparative Burning Characteristics of Solid Plastics in a Vertical Position.

[29] (2019). Standard Test Method for Measuring the Minimum Oxygen Concentration to Support Candle-Like Combustion of Plastics (Oxygen Index).

[30] (2015). Reaction-to-Fire Tests—Heat Release, Smoke Production and Mass Loss Rate—Part 1: Heat Release Rate (Cone Calorimeter Method) and Smoke Production Rate (Dynamic Measurement).

[31] Wang L.-S., Kang H.-B., Wang S.-B., Liu Y., Wang R. (2007). Solubilities, thermostabilities and flame retardance behaviour of phosphorus-containing flame retardants and copolymers. Fluid Phase Equilibria.

[32] Wei Z., Zhou C., Yu Y., Li Y. (2015). Poly(hexamethylene succinate) copolyesters containing phosphorus pendent group: Retarded crystallization and solid-state microstructure. Polymer.

[33] McAdam C.P., Hudson N.E., Liggat J.J., Pethrick R.A. (2008). Synthesis and characterization of nylon 6/clay nanocomposites prepared by ultrasonication and in situ polymerization. J. Appl. Polym. Sci..

[34] Sag J., Goedderz D., Kukla P., Greiner L., Schönberger F., Döring M. (2019). Phosphorus-Containing Flame Retardants from Biobased Chemicals and Their Application in Polyesters and Epoxy Resins. Molecules.

[35] Li Y., Lin Y., Sha K., Xiao R. (2016). Preparation and characterizations of flame retardant melamine cyanurate/polyamide 6 composite fibers via in situ polymerization. Text. Res. J..

[36] El Khatib W., Youssef B., Bunel C., Mortaigne B. (2003). Fireproofing of polyurethane elastomers by reactive organophosphonates. Polym. Int..

[37] Li J., Qian L., Xi W., Wang J., Qiu Y., Chen Y., Tang W. (2024). Alloying synergistic flame retardant effect on PA6 by polyimide containing alkyl hypophosphate structure. Eur. Polym..

[38] Zhang Y., Zheng W., Xiao Y., Yi C. (2023). Preparation of high-efficient flame retardant PA6 via DOPO-ITA initiated ring-opening polymerization of caprolactam. J. Polym. Sci..

[39] Ma C., Qiu S., Wang J., Sheng H., Zhang Y., Hu W., Hu Y. (2018). Facile synthesis of a novel hyperbranched poly(urethane-phosphine oxide) as an effective modifier for epoxy resin. Polym. Degrad. Stab..

[40] Carja I.-D., Serbezeanu D., Vlad-Bubulac T., Hamciuc C., Coroaba A., Lisa G., López C.G., Soriano M.F., Pérez V.F., Sánchez M.D.R. (2014). A straightforward, eco-friendly and cost-effective approach towards flame retardant epoxy resins. J. Mater. Chem. A.

[41] Schartel B., Hull T.R. (2007). Development of fire-retarded materials—Interpretation of cone calorimeter data. Fire Mater..

[42] Qian X., Guo N., Lu L., Wang X., Wang H., Shao G. (2018). Effect of Phosphorus-Based Flame Retardants and PA6 on the Flame Retardancy and Thermal Degradation of Polypropylene. Polym. Technol. Eng..

[43] Li Y., Li X., Pan Y.-T., Xu X., Song Y., Yang R. (2020). Mitigation the release of toxic PH3 and the fire hazard of PA6/AHP composite by MOFs. J. Hazard. Mater..

[44] Braun U., Schartel B. (2005). Effect of Red Phosphorus and Melamine Polyphosphate on the Fire Behavior of HIPS. J. Fire Sci..

