# Supplementary material for: Preparation of a Macromolecular Flame Retardant with a Phosphine Oxide Structure and Its Application in Polyamide 6
Source: Polymers (Basel). 2025 Feb 11;17(4):475. doi: 10.3390/polym17040475 (PMC11860063; doi:10.3390/polym17040475)
Supplement: Supplementary file 1 [file polymers-17-00475-s001.zip › polymers-3380339-supplementary.pdf]

Supplementary Materials: Preparation of a Macromolecular Flame Retardant with a Phosphine Oxide Structure and Its Application in Polyamide 6

Ke Liu [1,2,3,*], Bohan Liang [2,3], Shujuan Zhang [2], Ruyi Li [1], Junming Dai [2,3] and Wangyang Lu [1,2]

[1] National & Local Joint Engineering Research Center for Textile Fiber Materials and Processing Technology, School of Materials Science and Engineering, Zhejiang Sci-Tech University, Hangzhou, 310018, China; Ruyi_Li0311@163.com (R.L.); luwy@zstu.edu.cn (W.L.)
[2] Zhejiang Provincial Innovation Center of Advanced Textile Technology, Shaoxing, 312000, China; lbh20210319@163.com (B.L.); shujuanzhang@163.com (S.Z.); 13813128598@163.com (J.D.)
[3] Shaoxing Keqiao Research Institute of Zhejiang Sci-Tech University, Shaoxing, 312000, China
* Correspondence: liuke@zstu.edu.cn; Tel.: +86-571-86843611

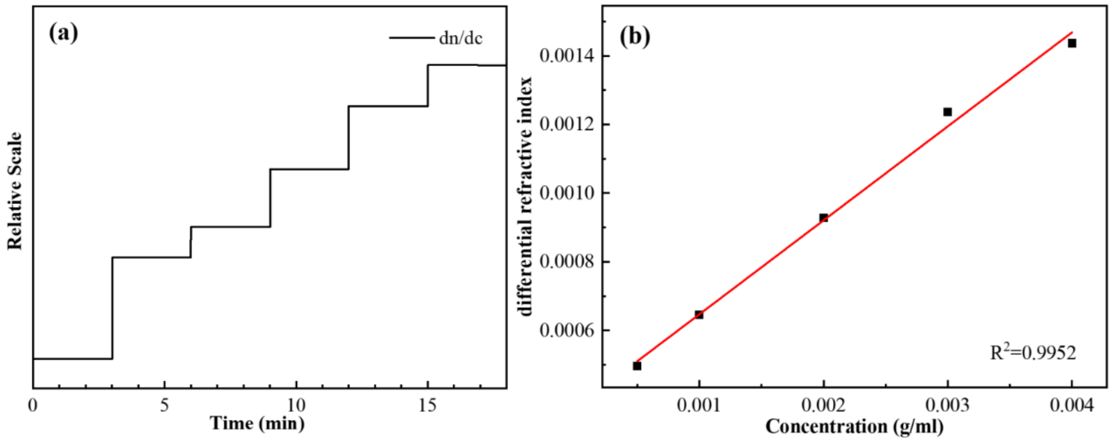

Figure S1. Concentration gradient curve of dn/dc (**a**) and refractive index standard curve of MFR (**b**).

The MFR sample solution, with a mass concentration of 4 mg·mL^{-1}, was prepared using HFIP containing 5 mmol·L^{-1} sodium trifluoroacetate as the solvent. The dn/dc values of the samples were determined using an injector coupled with a refractive index detector (RID). The Asrra 6 data acquisition and processing software was employed to measure dn/dc, with experimental parameters appropriately configured. A concentration gradient curve of dn/dc and a standard curve of refractive index at varying mass concentrations were generated and are presented in Figure S1. The standard curve exhibited a strong correlation coefficient of 0.9952, indicating high linearity. This suggests that the measured dn/dc values are accurate and reliable. The dn/dc value of MFR in HFIP containing sodium trifluoroacetate was determined by the software to be 0.2738 mL·g^{-1}.

Table S1. Solubility properties of CEPPA and MFR in different solvents (25 °C).

Sample	Water	Ethanol	DMSO	DMF
CEPPA	N	N	Y	Y
MFR-10	N	N	N	N
MFR-20	N	N	N	N
MFR-30	N	N	N	N
MFR-40	N	N	N	N

Note: Y represents insoluble, N represents dissolution.

The dissolution properties of CEPPA and MFR in various solvents are summarized in Table S1. In aqueous and ethanolic solutions, both CEPPA and MFR exhibited limited solubility. Upon initial mixing (5 minutes), these compounds formed a white turbid suspension in water, with powder accumulation in the upper layer. After 1 hour of mixing,

phase separation became apparent, with the powdered material gradually settling to the bottom. Prolonged mixing durations (2 hours, 5 hours, and 12 hours) did not significantly enhance dissolution, as substantial solid residues remained at the container bottom. In contrast, when DMSO and DMF were employed as solvents, distinct dissolution behaviors were observed. CEPPA demonstrated complete dissolution within 1 hour, yielding a clear, transparent solution. However, MFR exhibited markedly different characteristics in these solvents, with the majority of solid powder accumulating at the bottom and a minor portion remaining suspended, resulting in a persistently turbid solution (over 12 hours). The results demonstrated that MFR exhibits significantly better solvent resistance compared to CEPPA.

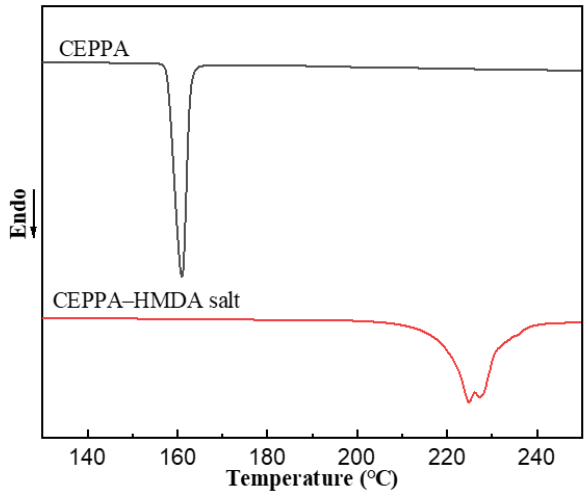

Figure S2. DSC heating curves of CEPPA and CEPPA–HMDA salt.

Figure S2 shows the DSC heating curves of CEPPA and CEPPA–HMDA salt, where the T_m of CEPPA was 159 °C. A single endothermic peak was observed for CEPPA–HMDA salt, indicating that the ammonification of CEPPA with HMDA yielded a CEPPA–HMDA salt, rather than a mixture of CEPPA and HMDA.

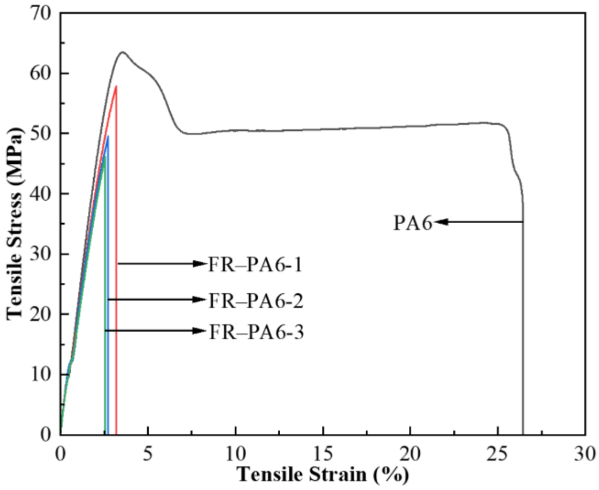

Figure S3. Stress-strain curves of PA6 and FR–PA6.

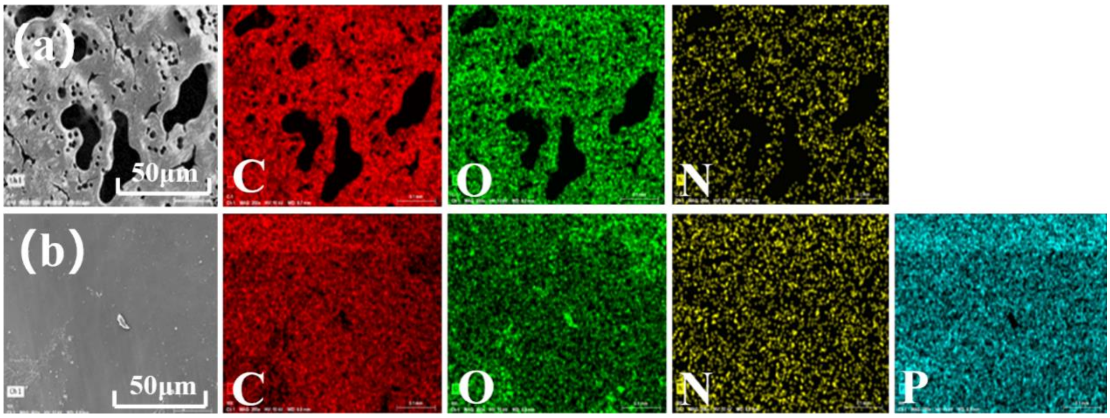

Figure S4. SEM-EDS images of PA6 (a) and FR–PA6-3 (b) carbon layer.

The SEM-EDS images of PA6 (a) and FR–PA6-3 (b) carbon layer were shown in Figure S4. The P element (3.23%) residue was detected in the energy spectrum of FR–PA6-3 carbon layer.
